# Lithium ionic conduction and relaxation dynamics of spark plasma sintered Li_5_La_3_Ta_2_O_12_ garnet nanoceramics

**DOI:** 10.1186/s11671-015-0777-7

**Published:** 2015-02-11

**Authors:** Mohamad M Ahmad

**Affiliations:** Department of Physics, College of Science, King Faisal University, Hofuf, Al-Ahsaa, 31982 Saudi Arabia; Physics Department, Faculty of Science, Assiut University in The New Valley, El-Kharga, The New Valley, 72511 Egypt

**Keywords:** Lithium garnet materials, Spark plasma sintering, Nanoceramics, Ionic conduction, Relaxation properties

## Abstract

In the present work, nanoceramics of Li_5_La_3_Ta_2_O_12_ (LLT) lithium ion conductors with the garnet-like structure are fabricated by spark plasma sintering (SPS) technique at different temperatures of 850°C, 875°C, and 900°C (SPS-850, SPS-875, and SPS-900). The grain size of the SPS nanoceramics is in the 50 to 100 nm range, indicating minimal grain growth during the SPS experiments. The ionic conduction and relaxation properties of the current garnets are studied by impedance spectroscopy (IS) measurements. The SPS-875 garnets exhibit the highest total Li ionic conductivity of 1.25 × 10^−6^ S/cm at RT, which is in the same range as the LLT garnets prepared by conventional sintering technique. The high conductivity of SPS-875 sample is due to the enhanced mobility of Li ions by one order of magnitude compared to SPS-850 and SPS-900 ceramics. The concentration of mobile Li^+^ ions, *n*_c_, and their mobility are estimated from the analysis of the conductivity spectra at different temperatures. *n*_c_ is found to be independent of temperature for the SPS nanoceramics, which implies that the conduction process is controlled by the Li^+^ mobility. Interestingly, we found that only a small fraction of lithium ions of 3.9% out of the total lithium content are mobile and contribute to the conduction process. Moreover, the relaxation dynamics in the investigated materials have been studied through the electric modulus formalism.

## Background

Li_5_La_3_Ta_2_O_12_ (LLT) lithium ion conductors with the garnet-like structure have received considerable research interests due to their electrical, electrochemical, and mechanical properties [1-13]. These materials have good ionicconductivity in the range of 10^−6^ S/cm at RT with small contributions from the grain boundaries and negligible electronic conductivity [1-3]. Lithium conducting garnets are also electrochemically stable in contact with lithium metal and other common electrodes and are also stable in ambient atmosphere [1-3]. These properties make LLT garnets promising alternatives for the hazards organic and polymer-based lithium electrolytes in lithium ion batteries produce. However, the recorded ionic conductivity values of LLT materials are much lower than the organic/polymer-based lithium electrolytes. Therefore, extensive research work is devoted to enhance the ionic conductivity in LLT conducting garnets. The most used strategy to enhance the conductivity is by chemical substitutions either by divalent cations on the La sites (such as Li_6_ALa_2_Ta_2_O_12_, A = Ba, Ca, Sr, Mg, [2-9]) or by trivalent cations on the Ta sites (such as Li_5+2x_La_3_Ta_2-x_Y_x_O_12_ [10]). By this strategy, the conductivity could reach a value of 10^−5^ to 10^−4^ S/cm at RT [2-10]. Moreover, the ionic conductivity of LLT garnets could be influenced by the preparation techniques (solid state reaction or sol-gel techniques) and processing conditions including the temperature of calcinations and sintering steps [1-11].

Another route to influence the ionic conductivity of ionic conducting materials is through modifying the microstructure. In several examples, the ionic conductivity increases for nanostructured materials such as CaF_2_-BaF_2_ fluoride ion conductors [14], CeO_2_ oxide ion conducting nanoceramics [15,16], and nanocrystalline LiNbO_3_ and LiTaO_3_ lithium ion conductors [17,18]. However, this is not straightforward since the ionic conductivity was found to decrease in other materials when the grain size is reduced [19]. Although nanocrystalline powder of LLT lithium garnet materials have been prepared by sol-gel techniques with a grain size of 100 to 200 nm, the grain size increases considerably to 3 μm after sintering the sample at 900°C for 5 h [11]. A similar behavior was also observed in Li_6_BaLa_2_Ta_2_O_12_ garnet materials [9]. The grain growth of the nanopowder is due to the conventional sintering which includes heating at high temperatures for long durations. Therefore, other innovative sintering techniques such as spark plasma sintering (SPS) could overcome these drawbacks and yield nanoceramic materials [15,16,20-23]. SPS experiments, which are performed at a lower temperature and for short time duration (within few minutes) compared to conventional sintering, can successfully minimize grain coarsening during the sintering process that leads to successful fabrication of dense nanomaterials. SPS is widely used to sinter a variety of conducting and non-conducting materials including lithium ion conductors, such as NASICON-type LiTi_2_(PO_4_)_3_ and LiHf_2_(PO_4_)_3_ [21,22], perovskite Li_3x_La_2/3-x_TiO_3_ [23], and garnet type Li_5_La_3_Bi_2_O_12_ lithium ion conductors [24].

In the current work, we synthesize the nanocrystalline Li^+^ ion conductor LLT by a combination of mechanical milling and solid state reaction techniques. The prepared nanopowder is sintered by spark plasma sintering at different temperatures. The microstructure, the ionic conduction, and relaxation properties will be studied in details.

## Methods

LLT was prepared by a combination of mechanical milling and solid state reaction techniques. Stoichiometric amounts of Li_2_CO_3_ (with 10 wt% excess of Li_2_CO_3_ was added to compensate for lithium loss at high temperatures), Ta_2_O_5_, and La_2_O_3_ (dried at 900°C overnight) were mixed together and calcinated at 700°C for 12 h. Before and after the calcination step, the powder was ball milled in 2-propanole for 12 h using tungsten carbide balls (10 mm diameter) and pots with a rotation speed of 350 rpm. The ratio of balls to powder mass was kept 10:1. The dried powder was then spark plasma sintered at different temperatures. In the SPS experiment, the product powder was sintered at 850°C, 875°C, and 900°C (call the samples SPS-850, SPS-875, and SPS-900, respectively) using SPS 4 - 10 system (4,000 amp, 10 tons: Thermal Technology LLC, Santa Rosa, USA). The SPS experiments were performed by using graphite die of 20 mm diameter under 60 MPa pressure with a heating rate of 100°C/min. The samples were first heated to 450°C and kept at this temperature for 5 min, and then the temperature was raised to the final sintering temperature. The dwelling time was fixed to 10 min followed by rapid cooling.

Powder X-ray diffraction and scanning electron microscopy measurements were performed for structural characterization of the materials. X-ray diffraction (XRD) data were collected over the 0 ≤ 2θ ≤ 100 range using a Stoe Stadi-P Image Plate, IP, (Stoe and Cie GmbH, Darmstadt, Germany), with monochromated Cu Kα1 radiation (λ = 1.5406 Å). Scanning electron microscopy (SEM) measurements were performed by JEOL SM7600F (JEOL Ltd., Akishima-shi, Japan) field emission scanning electron microscope in order to determine the grain size of the product materials. The electrical and relaxation properties were studied by impedance spectroscopy (IS) measurements performed on the sintered materials using Novocontrol concept 50 system in the 1 to 10^7^ Hz frequency range. The IS measurements were performed in the 200 to 400 K temperature range where the temperature was controlled by the Quatro cryosystem.

## Results and discussion

**Figure 1:**
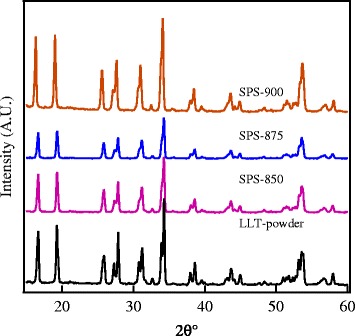
**XRD patterns of Li**_5_ **La**_3_ **Ta**_2_ **O**_12_ **powder and SPS nanoceramics.**

**Figure 2:**
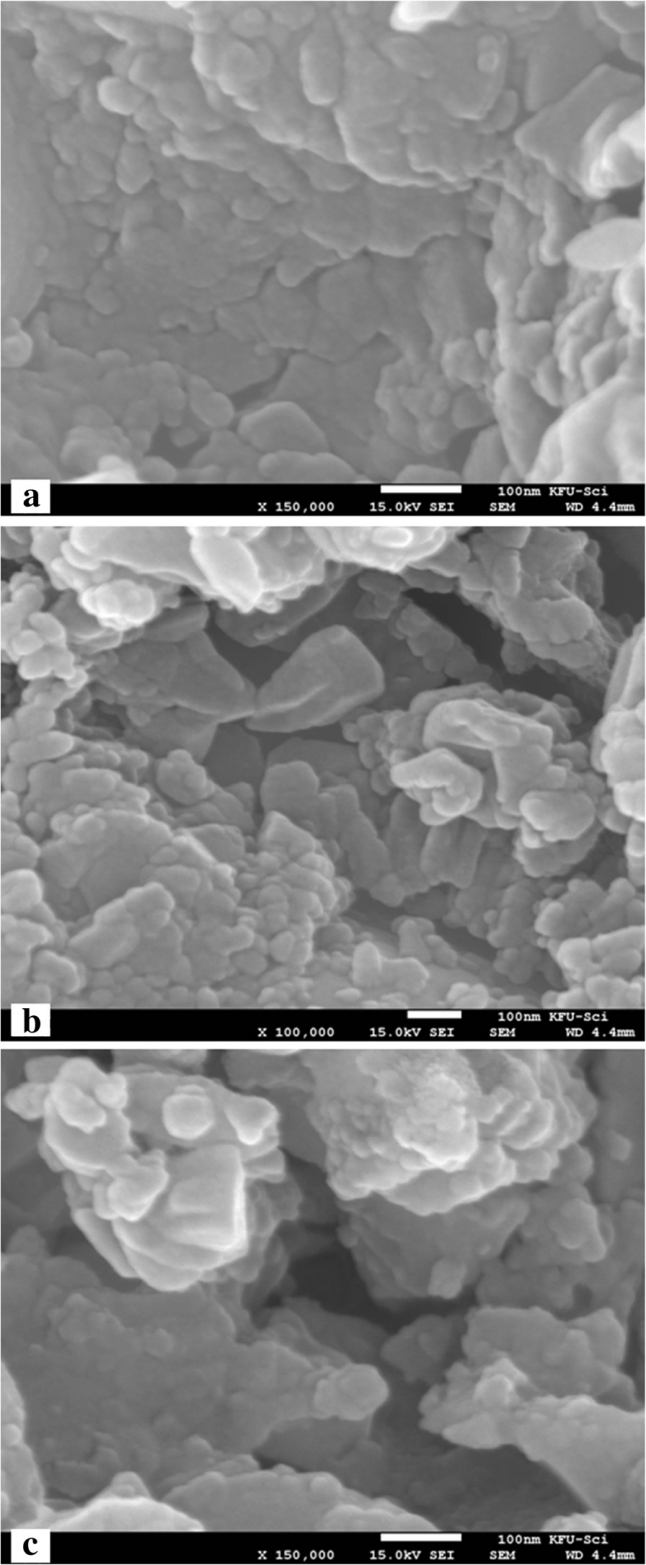
**SEM micrographs of SPS LLT nanoceramics. (a)** SPS-850, **(b)** SPS-875, and **(c)** SPS-900 LLT garnets. The bar in the figures is 100 nm.

X-ray powder diffraction patterns of the investigated materials are shown in Figure 1. All the spark plasma sintered samples show similar XRD patterns as the standard patterns of LLT with no secondary phases observed [1]. The SEM micrographs of the sintered ceramics are shown in Figure 2, which indicates that the SPS-850, SPS-875, and SPS-900 LLT ceramics have nano-sized grains with a grain size of 50 to 100 nm. These results indicate that spark plasma sintering produces nanoceramics of LLT garnet materials with considerably reduced grain size compared to the conventionally sintered ceramics that usually have coarse grained ceramics with grain size in the micrometer range [5,9,11].

**Figure 3:**
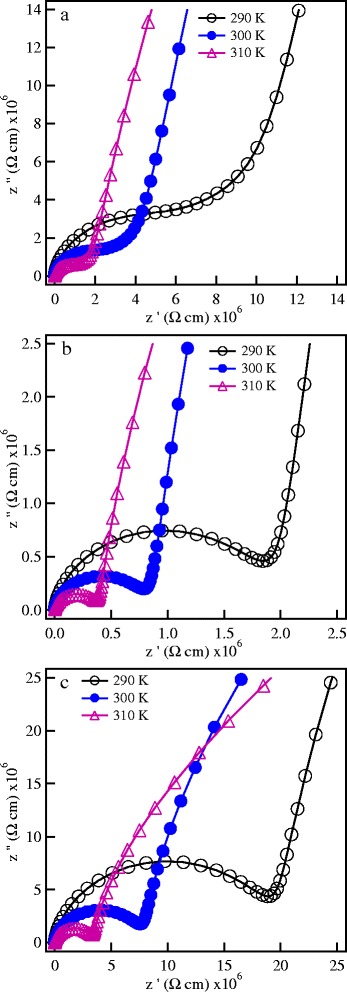
**Representative complex impedance diagrams at different temperatures of SPS LLT nanoceramics. (a)** SPS-850, **(b)** SPS-875, and **(c)** SPS-900 nanoceramics.

**Figure 4:**
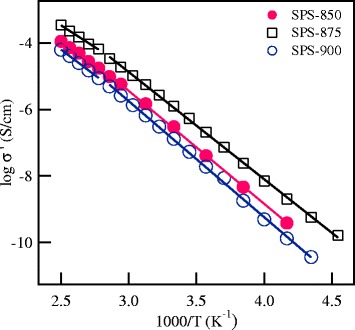
**The temperature dependence of the ionic conductivity for the SPS samples.**

The electrical properties of the investigated materials have been studied through impedance spectroscopy measurements. Representative complex impedance diagrams of the SPS ceramics are shown in Figure 3 at selected temperatures. The impedance diagrams show one semicircle at the high frequency region that could not be separated to grain and grain boundary contributions. Therefore, the intercept of the semicircle with the real axis represents the total (grain + grain boundary) ionic conductivity. At low frequencies, a large spike is observed which originates from electrode polarization effects and becomes more prominent at higher temperatures. The temperature dependence of the ionic conductivity of the SPS samples is shown in Figure 4. The values of the total conductivity at 27°C for the investigated materials are listed in Table 1. The total ionic conductivity first increases by one order of magnitude with increasing the SPS temperature from a value of 2.98 × 10^−7^ S/cm for the SPS-850 sample to 1.25 × 10^−6^ S/cm for the SPS-875 sample. With further increase of the SPS temperature to 900°C, the conductivity drops to 1.3 × 10^−7^ S/cm. The conductivity value of the SPS-875 nanoceramics in the present work is similar to the values reported previously for conventionally sintered LLT samples prepared either by solid state reaction or sol-gel techniques and sintered at 950°C and 900°C, respectively [1,11].

**Table 1:** **The conductivity and the activation energy of SPS LLT nanoceramics**

	***σ***_dc_ **(S/cm)**	**Δ** ***E*** **(e.V)**	**Δ** ***E*** **(e.V)**	***E***_σ_ **(e.V)**	***E***_H_ **(e.V)**	***E***_D_ **(e.V)**	***E***_m_ **(e.V)**
		**<350 K**	**>350 K**				
SPS-850	2.98 × 10^−7^	0.67	0.58	0.67	0.65	0.65	0.67
SPS-875	1.25 × 10^−6^	0.64	0.52	0.61	0.59	0.59	0.64
SPS-900	1.3 × 10^−7^	0.69	0.61	0.72	0.72	0.72	0.69

The dc conductivity σ_dc_ at 27°C and the associated activation energy Δ*E* at the low- and high-temperature regions of the SPS LLT garnet nanoceramics. The activation energy values determined from the conduction *E*_σ_, the hopping frequency *E*_H_, the diffusion coefficient *E*_D_, and the electric modulus relaxation time *E*_m_ are also summarized.

The conductivity data in Figure 4 shows two straight-line regions for the all studied samples with different values of activation energy. It is interesting mentioning that the conductivity data in the literature of most lithium conducting garnets are treated as a single straight line region with a single activation energy value, despite the clear curvature of the conductivity data especially at high temperatures [1-11]. Recent conductivity and NMR studies on Li_6.5_La_2.5_Ba_0.5_ZrTaO_12_ garnets showed two temperature regions with activation energy values of 0.57 and 0.37 e.V for the low- and high-temperature regions, respectively [25]. The conductivity data in Figure 4 could be fitted by the Arrhenius relation:

1 $$ \sigma = {\sigma}_o\  \exp\ \left( - \frac{\varDelta E}{k\ T}\right), $$

where *σ*_o_ is the pre-exponential factor, *k* is Boltzman constant, and Δ*E* is the activation energy for the ionic conduction. The values of the activation energy determined from the conductivity data are summarized in Table 1. It is noticed from this table that the activation energy values of the low-temperature region (<350 K) are higher than in the high-temperature region, with the SPS-875 sample showing the lowest activation energy. The presence of two thermally activated regions may indicate a change in the conduction mechanism with increasing temperature [25].

In order to understand the conduction behavior of the investigated materials, it is essential to determine the primary factors that control the ionic conduction process. The dc conductivity could be described by the following relation:

2 $$ {\sigma}_{dc}=e\ {n}_c\ \mu $$

where *e* is the electronic charge, *n*_*c*_ is the concentration of mobile charge carriers, and *μ* is the charge carriers' mobility. Therefore, the primary factors that influence the ionic conduction process are the concentration and mobility of Li^+^ ions. Here, we can estimate the concentration of the mobile Li^+^ ions *n*_c_ and their mobility *μ* through the analysis of the frequency dependence of the real part of the complex conductivity. The conductivity spectra of different ionic conductors are usually analyzed by a power-law model of the form [26],

3 $$ \sigma\;\hbox{'}\left(\omega \right)\kern0.5em =\kern0.5em {\sigma}_{dc}\;\left[1+{\left(\omega /{\omega}_c\right)}^n\right], $$

where *ω* is the angular frequency, *n* is the power-law exponent, and *ω*_*c*_ is the crossover frequency from the dc to the dispersive conductivity region. The dc conductivity in Equations 2 and 3 could be given by the Nernst-Einstein relation;

4 $$ {\sigma}_{dc}\kern0.5em =\kern0.5em e{n}_c\mu \kern0.5em =\kern0.5em \frac{n_c\;{e}^2\;\gamma\;{\lambda}^2}{k\;T}\;{\omega}_{\mathrm{H}}, $$

where *γ* is a geometrical factor for ion hopping, *λ* is the hopping distance, and *ω*_H_ is the hopping frequency of mobile ions. The crossover frequency *ω*_c_ represents a good estimate of the true hopping frequency of mobile ions, *ω*_H_ [27-30]. Therefore, we have analyzed the conductivity spectra of the investigated materials using Equation 3 in order to determine the values of *σ*_*dc*_ and *ω*_c_.

**Figure 5:**
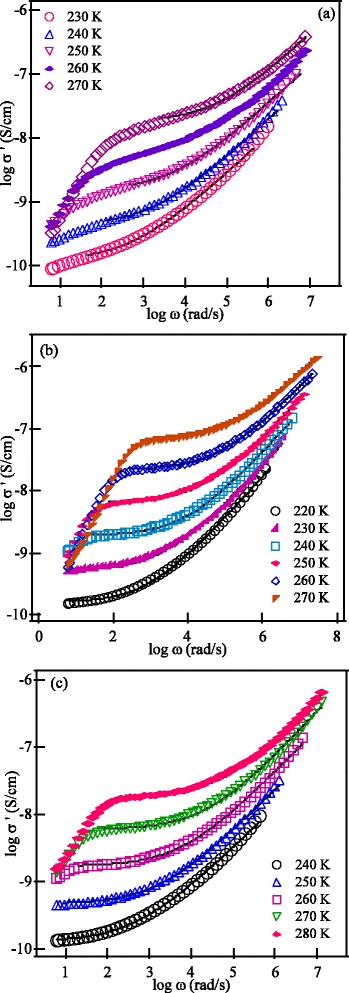
**The conductivity spectra at different temperatures. (a)** SPS-850, **(b)** SPS-875, and **(c)** SPS-900 samples. The solid curves are the best fits according to Equation 3.

**Figure 6:**
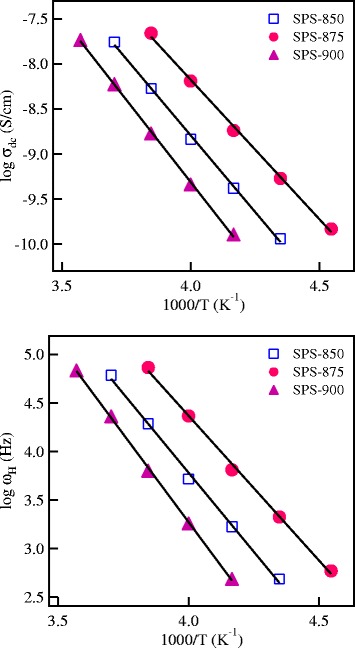
**The temperature dependence of** ***σ***_dc_ **and** ***ω***_H_ **of the SPS lithium conducting garnets.** *σ*_dc_ and *ω*_H_ have been determined from the fitting of the conductivity spectra in Figure 5.

The fitting results of the conductivity data are shown as solid curves in Figure 5a,b,c for SPS-850, SPS-875, and SPS-900 garnet nanoceramics. The extracted values of *σ*_*dc*_, *ω*_H_ and *n* are summarized in Table 2, and the temperature dependence of *σ*_*dc*_ and *ω*_H_ is shown in Figure 6. Both *n*_c_ and *ω*_H_ of mobile Li^+^ ions may be thermally activated and could be written as [28]

**Table 2:** **The parameters of the conduction process in SPS LLT garnet nanoceramics**

**T (K)**	***σ***_dc_ **(S/cm)**	***ω***_H_ **(Hz)**	***n***	***n***_c_ **(cm** ^−3^ **)**	***μ*** **(cm** ^2^ **V** ^−1^ **s** ^−1^ **)**	***D*** **(cm** ^2^ **s** ^−1^ **)**
SPS-850						
230	1.15 × 10^−10^	4.87 × 10^2^	0.60	6.08 × 10^20^	1.18 × 10^−12^	2.34 × 10^−14^
240	4.16 × 10^−10^	1.68 × 10^3^	0.60	6.62 × 10^20^	3.92 × 10^−12^	8.11 × 10^−14^
250	1.45 × 10^−9^	5.21 × 10^3^	0.60	7.78 × 10^20^	1.16 × 10^−11^	2.51 × 10^−13^
260	5.32 × 10^−9^	1.93 × 10^4^	0.61	7.99 × 10^20^	4.16 × 10^−11^	9.31 × 10^−13^
270	1.75 × 10^−8^	6.10 × 10^4^	0.62	8.65 × 10^20^	1.26 × 10^−10^	2.94 × 10^−12^
SPS-875						
220	1.47 × 10^−10^	5.90 × 10^2^	0.61	6.13 × 10^20^	1.50 × 10^−12^	2.84 × 10^−14^
230	5.37 × 10^−10^	2.12 × 10^3^	0.61	6.50 × 10^20^	5.16 × 10^−12^	1.02 × 10^−13^
240	1.83 × 10^−9^	6.48 × 10^3^	0.61	7.56 × 10^20^	1.51 × 10^−11^	3.12 × 10^−13^
250	6.44 × 10^−9^	2.32 × 10^4^	0.62	7.74 × 10^20^	5.19 × 10^−11^	1.12 × 10^−12^
260	2.18 × 10^−8^	7.33 × 10^4^	0.62	8.64 × 10^20^	1.57 × 10^−10^	3.53 × 10^−12^
SPS-900						
240	1.28 × 10^−10^	4.88 × 10^2^	0.60	7.02 × 10^20^	1.14 × 10^−12^	2.35 × 10^−14^
250	4.63 × 10^−10^	1.83 × 10^3^	0.62	7.07 × 10^20^	4.09 × 10^−12^	8.80 × 10^−14^
260	1.69 × 10^−9^	6.36 × 10^3^	0.64	7.71 × 10^20^	1.37 × 10^−11^	3.06 × 10^−13^
270	5.97 × 10^−9^	2.30 × 10^4^	0.66	7.81 × 10^20^	4.77 × 10^−11^	1.11 × 10^−12^
280	1.85 × 10^−8^	6.83 × 10^4^	0.64	8.45 × 10^20^	1.37 × 10^−10^	3.30 × 10^−12^

The values of the dc conductivity *σ*_dc_, the hopping frequency *ω*_H_, the exponent *n*, the concentration of mobile Li ions *n*_c_, the mobility *μ*, and the diffusion coefficient *D* are summarized for the SPS LLT garnet nanoceramics at different temperatures.

5a $$ {n}_c = {n}_0\ Exp\ \left( - \frac{E_c}{kT}\ \right), $$

5b $$ {\omega}_H = {\omega}_0\ Exp\ \left( - \frac{E_H}{kT}\ \right), $$

where *E*_c_ and *E*_H_ are the activation energies for the creation and migration of charge carriers, respectively. It is observed from Equations 4 and 5 that the activation energy of the dc conductivity is *E*_σ_ = *E*_c_ + *E*_H_. The activation energy values for the ionic conduction, *E*_σ_, and for ion hopping, *E*_H_, determined from the straight-line fits of the data in Figure 6 are listed in Table 1 for the SPS garnet nanoceramics. The close agreement of *E*_σ_ and *E*_H_ leads to a value of *E*_c_ ~ 0 e.V, indicating that the concentration of mobile Li^+^ ions, *n*_c_, is independent of temperature [27-30]. Since *n*_c_ is independent of temperature for each SPS sample, then the ionic conduction in these materials is controlled by the mobility of mobile Li^+^ ions.

The values of *n*_*c*_ for mobile Li^+^ ions in LLT nanoceramics have been estimated using Equation 4, with a hopping distance as short as *λ* = 1.7 Å has been used [31]. The estimated values of *n*_c_ at different temperatures for the investigated materials are listed in Table 2. We notice that the values of *n*_c_ for all the SPS LLT ceramics are almost independent of temperature with average values of 7.42 × 10^20^, 7.31 × 10^20^, and 7.61 × 10^20^ cm^−3^ for SPS-850, SPS-875, and SPS-900 nanoceramics, respectively. These results show that the values of *n*_c_ for all the SPS LLT garnets are almost the same. Therefore, the enhanced conductivity of SPS-875 ceramics by about one order of magnitude compared to SPS-850 and SPS-900 samples is due to the enhanced Li ionic mobility/hopping frequency as observed in Figure 6b and Table 2. There are different factors that may affect the mobility of Li ions in garnet materials processed by spark plasma sintering including the grain size, the grain-to-grain bonding, the possible loss of Li at high sintering temperatures, and the possible formation of secondary insulating phases in the materials [20]. The enhanced mobility/conductivity in SPS-875 ceramics compared to SPS-850 could be due to the improved grain-to-grain bonding with increasing the SPS temperature [20-23]. With further increasing the SPS temperature, partial decomposition and/or loss of Li could occur, which may lead to the formation of minor impurity phases in the materials [20,32,33]. These features could be the reasons for the drop of the conductivity for SPS-900 ceramics. Similar dependence of the Li ionic conductivity on the SPS temperature was observed for Li_5_La_3_Nb_2_O_12_ and Li_1.3_Al_0.3_Ti_1.7_(PO_4_)_3_ ceramics [33,34].

It is interesting to compare the values of *n*_c_ with that of the total density *N* of Li^+^ ions, which is calculated using the relation; *N* = *m*/*V*, where *m* is the number of lithium ions per unit cell (*m* = 40 in LLT) and *V* is the volume of the unit cell. Using a lattice parameter value of 12.804 Å [1] gives a value of *N* of 1.91 × 10^22^ cm^−3^. Accordingly, the ratio of the concentration of mobile Li^+^ ions, *n*_c_, to the total Li^+^ density, *N*, is about 3.9% in the spark plasma sintered LLT nanoceramics.

The diffusion coefficient, *D*, of Li^+^ ions could be estimated from the following relation:

6 $$ {\sigma}_{dc} = \frac{e^2\ {n}_c\ }{k\ T}\ D $$

**Figure 7:**
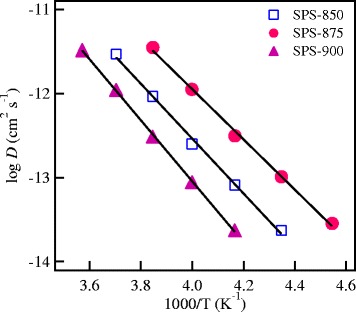
**The temperature dependence of the diffusion coefficient** ***D*** **of the SPS garnets.**

The values of the mobility *μ* and the diffusion coefficient *D* of mobile Li^+^ ions in the SPS LLT garnets have been calculated using Equations 2 and 6, respectively. The values of *μ* and *D* at different temperatures are listed in Table 2. Moreover, the temperature dependence of *D* for different SPS LLT nanoceramics is shown in Figure 7. The diffusion coefficient is thermally activated with the same activation energy of the conduction process as is observed in Table 1. The extrapolation of the diffusion coefficient to RT (27°C) gives a value of *D* of 4.36 × 10^−11^, 1.11 × 10^−10^, and 2.37 × 10^−11^ cm^2^ s^−1^ for SPS-850, SPS-875, and SPS-900 samples, respectively. These values of *D* are two to three orders of magnitude lower than Li superionic conductors (*D* ~ 10^−8^ cm^2^ s^−1^) that exhibit ionic conductivity values of >10^−3^ S/cm at RT [35].

**Figure 8:**
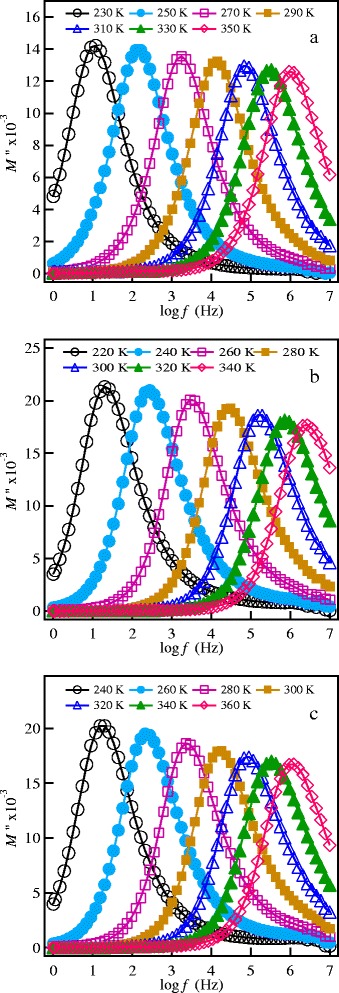
**The electric modulus spectra at different temperatures. (a)** SPS-850, **(b)** SPS-875, and **(c)** SPS-900 garnet nanoceramics.

**Figure 9:**
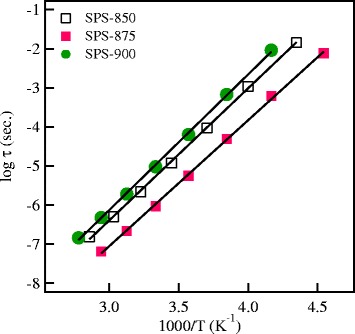
**The temperature dependence of the conductivity relaxation time for the SPS garnets.**

The relaxation dynamics of Li^+^ ions in SPS LLT nanoceramics are studied through the frequency dependence of the electric modulus formalism. The electric modulus is related to the impedance by the relation:

7 $$ {M}^{*}\left(\omega \right)=j\omega {C}_o{Z}^{*}\left(\omega \right) $$

where *C*_o_ = *ε*_o_*A*/*d* is the capacitance of a free sample cell with electrode aria *A* and electrode separation *d*, and *ε*_o_ = 8.854 × 10^−14^ F/cm is the permittivity of free space. The frequency dependence of the imaginary part of the electric modulus, *M''*, at different temperatures is shown in Figure 8a,b,c for the investigated garnet materials. Well-defined peaks are observed in the modulus spectra. These peaks represent re-orientation relaxation process of mobile Li^+^ ions [10]. The low-frequency side of the peaks is the region where Li^+^ ions are capable to form successful hopping from one site to the next, whereas the high frequency side of the peak is where Li^+^ ions can perform local (re-orientation) motion only [36]. The peak positions in the modulus spectra shift toward high frequency with increasing temperature, indicating a thermally activated relaxation process [36]. The most probable conductivity relaxation time is determined from the frequency of the peak according to the relation:

*τ*_*M*_ = 1/2*πf*_max_, where *f*_max_ is the frequency at peak maximum. The temperature dependence of the relaxation time of the investigated materials is presented in Figure 9 and could be expressed in the following Arrhenius relation:

8 $$ \tau = {\tau}_o \exp \left(\frac{E_m}{kT}\right) $$

where *τ*_o_ is the pre-exponential factor and *E*_m_ is the activation energy of the relaxation process. The values of *E*_m_ are summarized in Table 1 and agree with the activation energy of the ionic conduction process in the low-temperature region. This agreement indicates that mobile Li^+^ ions are responsible for both the long-range transport and re-orientation relaxation process in the garnet materials.

**Figure 10:**
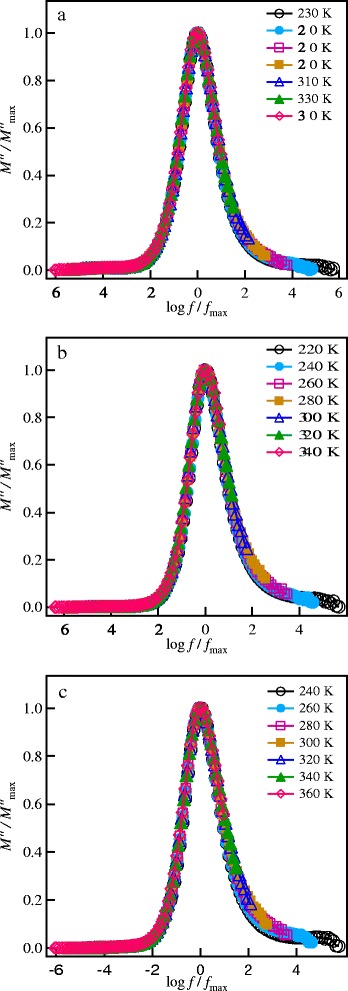
**Scaling of the modulus spectra at different temperatures. (a)** SPS-850, **(b)** SPS-875, and **(c)** SPS-900 garnets.

**Figure 11:**
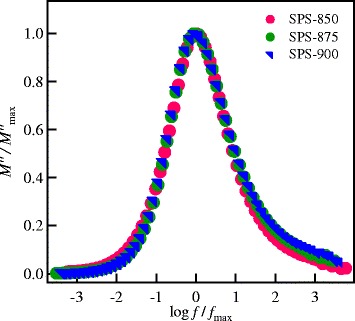
**Scaling of the modulus spectra for all SPS garnet nanoceramics at different temperatures.**

The modulus scaling of the investigated SPS garnets have been performed using ${M}_{\max}^{\prime \prime }$ and *f*_max_ as the scaling parameters of the *M*′ and the frequency axes, respectively. The results of the scaling processes at different temperatures for the SPS LLT ceramics are shown in Figure 10. The modulus spectra at different temperatures are superimposed into a single master curve for each sample, indicating that the relaxation process is independent of temperature. The full width at half maximum (FWHM) has similar values of 1.77 decades for the SPS-850, SPS-875, and SPS-900 nanoceramics. These values are larger than that of the ideal Debye relaxation (FWHM of 1.14 decades, ref. [37]), which suggests a distribution of relaxation times due to disordering nature in the current lithium garnet nanoceramics. Scaling of the modulus spectra of the different SPS ceramics together at different temperatures is shown in Figure 11. This figure shows that the modulus spectra of all the samples are merged into a single master curve, implying that the studied materials exhibit similar relaxation processes.

## Conclusions

The present study confirmed that spark plasma sintering is a powerful technique to fabricate nanoceramics of different types of materials including lithium conducting garnets. Nanoceramics of Li_5_La_3_Ta_2_O_12_ lithium conducting garnets with grain size of 50 to 100 nm have been achieved by SPS experiments at 850°C, 875°C, and 900°C for a short dwelling time of 10 min. SPS-875 sample shows the highest ionic conductivity of 1.25 × 10^−6^ S/cm at RT. The ionic conductivity exhibits two temperature regions with different activation energy, which suggests changing the conduction mechanism at high temperatures. The parameters that usually control the ionic conduction, the concentration and mobility of mobile ions, have been estimated. The concentration of mobile Li^+^ ions is independent of temperature; therefore, the enhanced conductivity is attributed to the enhanced mobility of Li^+^ ions. The fraction of Li^+^ ions that is mobile and participates in the conduction process surprisingly represents a small percentage of only 3.9% out of the total density of Li content in the current LLT garnets. The relaxation processes in the SPS garnet nanoceramics are found to be independent of temperature, and the conduction and relaxation processes are thermally activated by the same activation energy, which implies that Li^+^ ions are the origin of both the long-range transport as well as the short-range (local) reorientation relaxation in the garnet materials.
